# Finger Nails

**Published:** 1882-07

**Authors:** 


### FINGER NAILS.

The finger nails grow their whole length in a few months —faster in summer than in winter. They should be trimmed once a week slowly and composedly, with a pair of sharp blunt-end scissors, closer at the ends than at the sides, thus preventing the skin from rising, causing as to the toes, that most distressing ailment called in-growing toe nail. Accumulations under the ends of the nails should not be removed by any metallic substance, but by a piece of soft wood, or which is still better, by a thorough washing of the hands. We have seen "beauty and the beast" united, in a handsome face and a suggestive rim of black at the end of the fingers

### A FILTHY ATMOSPHERE.

We were passing a corner on two prominent streets the other evening, where there were assembled perhaps a dozen ladies and gentlemen waiting for the cars, when our nostrils were assailed with the most fearful stench of an atmosphere, to be found anywhere outside of a class-room in one of our public schools. Looking around for the cause, we found it to be the air still adhering to the clothing of these people who had just come from one of the negro-minstrel shops, where they had been spending the evening.

